# Up-Regulation of *FOXC2* and *FOXQ1* Is Associated with The Progression of Gastric-Type Adenocarcinoma

**DOI:** 10.22074/cellj.2017.4357

**Published:** 2017-05-17

**Authors:** Farzad Soleimani, Mohammadreza Hajjari, Bahram Mohammad Soltani, Mehrdad Behmanesh

**Affiliations:** 1 Department of Genetics, Faculty of Biological Sciences, Tarbiat Modares University, Tehran, Iran; 2 Department of Genetics, Faculty of Sciences, Shahid Chamran University of Ahvaz, Ahvaz, Iran

**Keywords:** Gastric Cancer, Gene Expression, *FOXC2*, *FOXQ1*, Quantitative Polymerase Chain Reaction

## Abstract

**Objective:**

Forkhead box (FOX) proteins are important regulators of the epithelial-to-mesenchymal transition (EMT), which is the main mechanism of cancer metastasis. Different studies have shown their potential involvement in progression of cancer in different tissues such as breast, ovary and colorectum. In this study, we aimed to analyze the expression of genes encoding two FOX proteins in gastric adenocarcinoma.

**Materials and Methods:**

In this experimental case-control study, the expression of *FOXC2* and *FOXQ1* was examined in 31 gastric adenocarcinoma tumors and 31 normal adjacent gastric tissues by reverse transcription polymerase chain reaction (PCR).

**Results:**

The expression of both genes was significantly up-regulated in gastric adenocarcinoma tumors compared with the normal tissues (P<0.05). The differential expression of these two genes was also correlated with the grade of tumors (P<0.01).

**Conclusion:**

We show that up-regulation of *FOXC2* and *FOXQ1* are likely to be involved in the progression of gastric adenocarcinoma.

## Introduction

Gastric cancer is one of the most common malignancies worldwide with a high mortality rate. Interestingly, the vast majority of gastric cancers are adenocarcinomas. Therefore, an improved etiologic understanding of this cancer type seems to be essential. Although some classic and novel biomarkers such as carcinoembryonic antigen (CEA) and cancer antigen 19-9 (CA19- 9) (1) are known, finding effective biomarkers for early detection is yet to be identified. Also, developing new therapeutic options for this type of cancer is a necessity (2). The forkhead box (*FOX*) gene family encodes a large and diverse group of transcription factors. These factors share specific characteristics such as a conserved 100 amino acid long DNA-binding domain known as the forkhead or winged helix domain (3). There are 17 *FOX* subfamilies (*FOXA-R*) with a total of 41 genes currently identified in humans (4). Despite the high sequence conservation in the core forkhead motifs, *FOX* proteins control different cell fate decisions by regulating different gene networks involved in cell cycle progression, proliferation and differentiation. They contribute to different processes such as metabolism, senescence, survival and apoptosis (5). Multiple studies have therefore reported these genes and have shown their role in diseases such as cancer (6).

*FOXC2*, a member of the *FOX* family, is shown to be an essential regulator of vascular/ lymphatic vessel formation in cardiovascular development and disease (7). Furthermore, *FOXC2* expression is directly associated with the epithelial mesenchymal transition (EMT) and cancer stem cell properties in breast cancer (1, 8). This gene has been also shown to be up-regulated in other cancers including those of the colorectum, glial cells and cervix (9-11). Independent studies have proposed that the encoded protein is involved in EMT of cancer cells, a critical process in tumor genesis and metastasis (10, 11). The studies have also described a key role for another member of the *FOX* family, namely forkhead box Q1 (*FOXQ1*), in regulating EMT and aggressiveness in some human cancers (12, 13). It has also been shown that this gene is aberrantly expressed in colorectal, breast, lung, glial cell and gastric cancers (3, 12, 14, 15). However, the clinical significance of *FOXQ1* expression level in gastric adenocarcinoma has not been confirmed by an independent study. Due to the important role of EMT in the progression of gastric cancer (16, 17), we aimed to analyze the expression of these two genes in gastric adenocarcinoma. We suggest that protein encoded by these two genes are potentially involved in the clinicopathology of gastric adenocarcinoma tumors.

## Materials and Methods

A total of 62 samples comprising 31 gastric adenocarcinoma tumors and their normal adjacent gastric tissues were obtained from Iran National Tumor Bank (Iran). Clinical features of the donor patients are given in Table 1. All patients had not received any medication prior. Also, all had signed a written informed consent prior to surgery and all specimens were evaluated by two pathologists. The tumor samples were grouped under two category of grade [low grade (n=17) and high grade (n=14)] and stage [T1-T2 (n=10) and T3-T4 (n=21)]. The design of the experiment was approved by the Medical Ethics Committee of Tarbiat Modares University. All tissue specimens were frozen in liquid nitrogen after collection and then stored in -80˚C.

### Total RNA extraction and cDNA synthesis

Total RNA was extracted by acid guanidinium-phenol-chloroform procedure using the RNX™-plus solution (CinnaGen, Iran) according to the manufacturer’s protocol. The quality and integrity of extracted RNA were assessed by gel electrophoresis (1% agarose) and spectrophotometry respectively. To remove genomic DNA contamination, total RNA was treated with DNase I (Sigma, USA) at 37˚C for 30 minutes. Three microgram of total RNA was reverse transcribed with oligo dT and random hexamers (MWG, Germany) by RevertAidTM Reverse Transcriptase (Fermentas, Canada) in a total volume of 20 μl according to the manufacturer’s protocols.

**Table 1:** Clinical features of donor patients with gastric adenocarcinoma


Clinical parameter	Number of individuals

Samples	31
Gender		
Male	18
Female	13
Age
<64	15
≤64	16
Tumor site
Cardia	10
Non cardia	21
Grade of tumors
Low grade	17
High grade	14
Staging
T1-T2	10
T3-T4	21
Lymphatic invasion
Yes	18
No	13
Preineural invasion
Yes	9
No	22


### Real-time quantitative polymerase chain reaction

Expression of *FOXQ1* and *FOXC2* transcripts was quantified by reverse-transcription quantitative polymerase chain reaction (RT-qPCR) technique by a 7500 Real Time PCR System (Applied Biosystems, Foster City, CA, USA). The PCR primers were designed using the Allele ID software and are given in Table 2. The expression of each transcript was normalized with glyceraldehyde 3-phosphate dehydrogenase (*GAPDH*) transcripts as an internal control. The reaction mixture consisted of 10 ng cDNA, 10 μl of 2X SYBR Green I master mix (Takara, Japan) and 200 nM of forward and reverse primers in a total volume of 20 μl according to the manufacturer’s instructions. The cycling conditions were an initial denaturation for 30 seconds at 94˚C, followed by 40 cycles of denaturation at 95˚C for 10 seconds and annealing/ extension at 60˚C for 30 seconds. All reactions of q-PCR were run in triplicate. The specificity of the PCR products was examined by melting curve analysis, followed by electrophoresis on a 12% polyacrylamide gel and digestion pattern. In order to obtain the standard curve for each primer set, a serially diluted cDNA sample was used. The Livak method (ΔΔCT) was used to analyze differential gene expression (18).

**Table 2:** Sequence of primers used for real time polymerase chain reaction (PCR)


Gene	Primer sequence (5ˊ-3ˊ)	PCR product length

*FOXQ1*	F: TGCTATTGACCGATGCTTCAC	152
	R:CCAAGGAGACCACAGTTAGAG	
*FOXC2*	F: CGGCCCAGCAGCAAACTTTCC	139
	R:AGAGGCGGCGTGGATCTGTAG	
GAPDH	F: CCATGAGAAGTATGACAAC	115
	R: GAGTCCTTCCACGATACC	


### Statistical analysis

Statistical analysis was based on paired Student’s t test. Categorical data including grade and stage of tumors were analyzed by t test through GraphPad Prism software. Pearson correlation coefficient was also calculated between normalized expressions of both genes. The P<0.05 were considered as significant. Multiple testing corrections were done by Bonferroni adjustment procedure.

## Results

### *FOXC2* and *FOXQ1* are up-regulated in gastric adenocarcinoma

**Fig.1:**
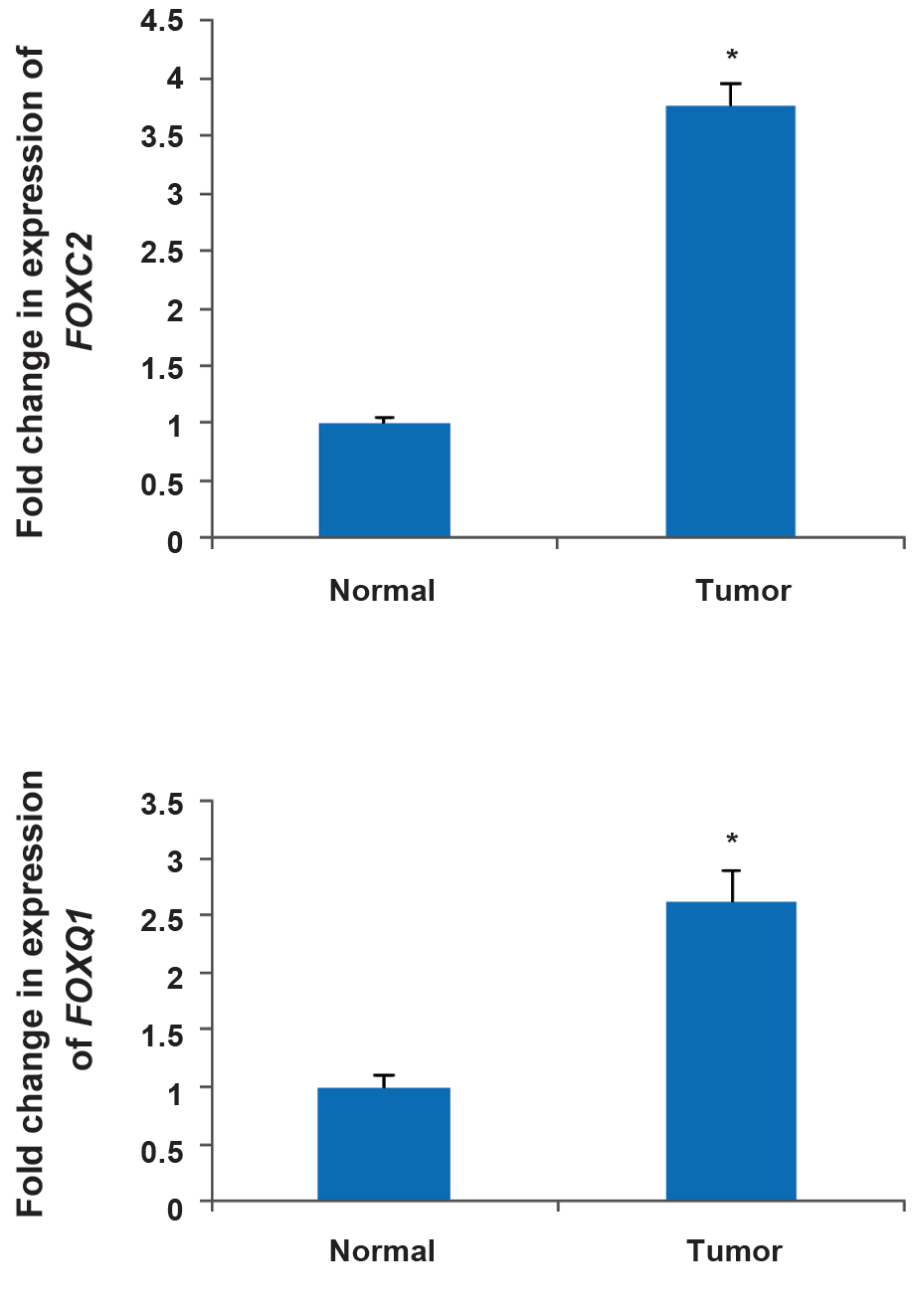
The up-regulation of *FOXC2* and *FOXQ1* in gastric adenocarcinoma tumors compared with normal tissues. The results are achieved by real time polymerase chain reaction (PCR). *; P<0.05.

Gene expression analysis showed that *FOXC2* and *FOXQ1* transcript levels were significantly higher in gastric adenocarcinoma samples than in normal pair tissues. The average fold-change for *FOXC2* and *FOXQ1* expression levels in gastric adenocarcinoma samples was 3.076 (P=0.045) and 2.622 (P=0.03) respectively (Fig.1). However, Bonferroni correction did not find them as significant difference. Furthermore, correlation analysis showed that the normalized expression of *FOXC2* and *FOXQ1* are correlated in tumors compared to normal tissues (r=0.436, P<0.05).

### *FOXC2* and *FOXQ1* have higher expression in high-grade tumors compared with low-grade tissues

**Fig.2:**
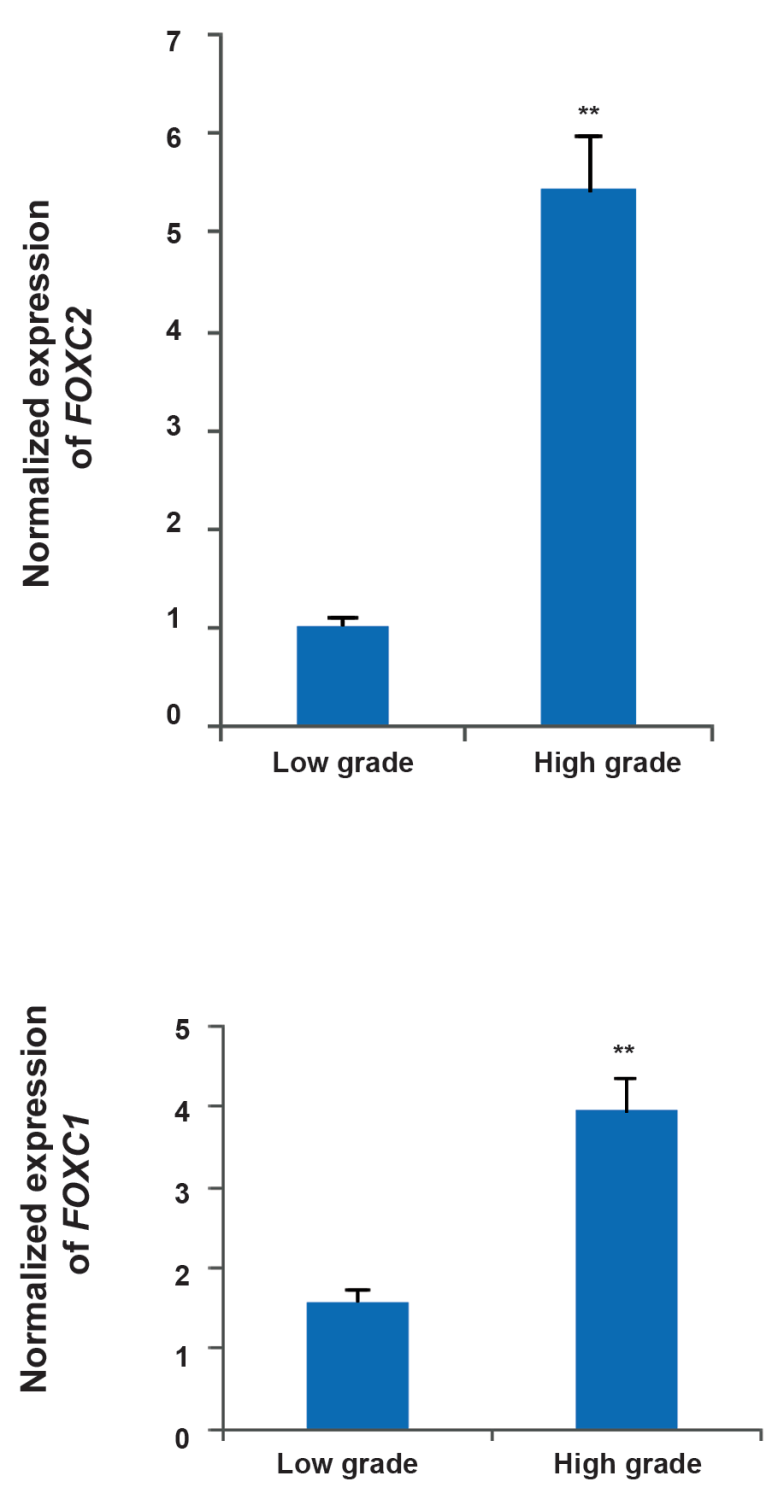
The significant difference of expression level of *FOXC2* and *FOXQ1* between tumors with different grades. The results show that the genes are up-regulated in high-grade tumors. The results are achieved by real time polymerase chain reaction (PCR). **; P<0.01.

In order to determine any potential association between the expression levels of either gene and progression of gastric tumor, we compared their expression levels between tumors of low and high grades. We found that *FOXC2* and *FOXQ1* are significantly over-expressed in high-grade tumors with fold changes of 5.44 and 3.953 respectively (Fig.2, P<0.01). Multiple testing also showed them as significant.

### *FOXC2* and *FOXQ1* expression analysis in high and low stage tumors

**Fig.3:**
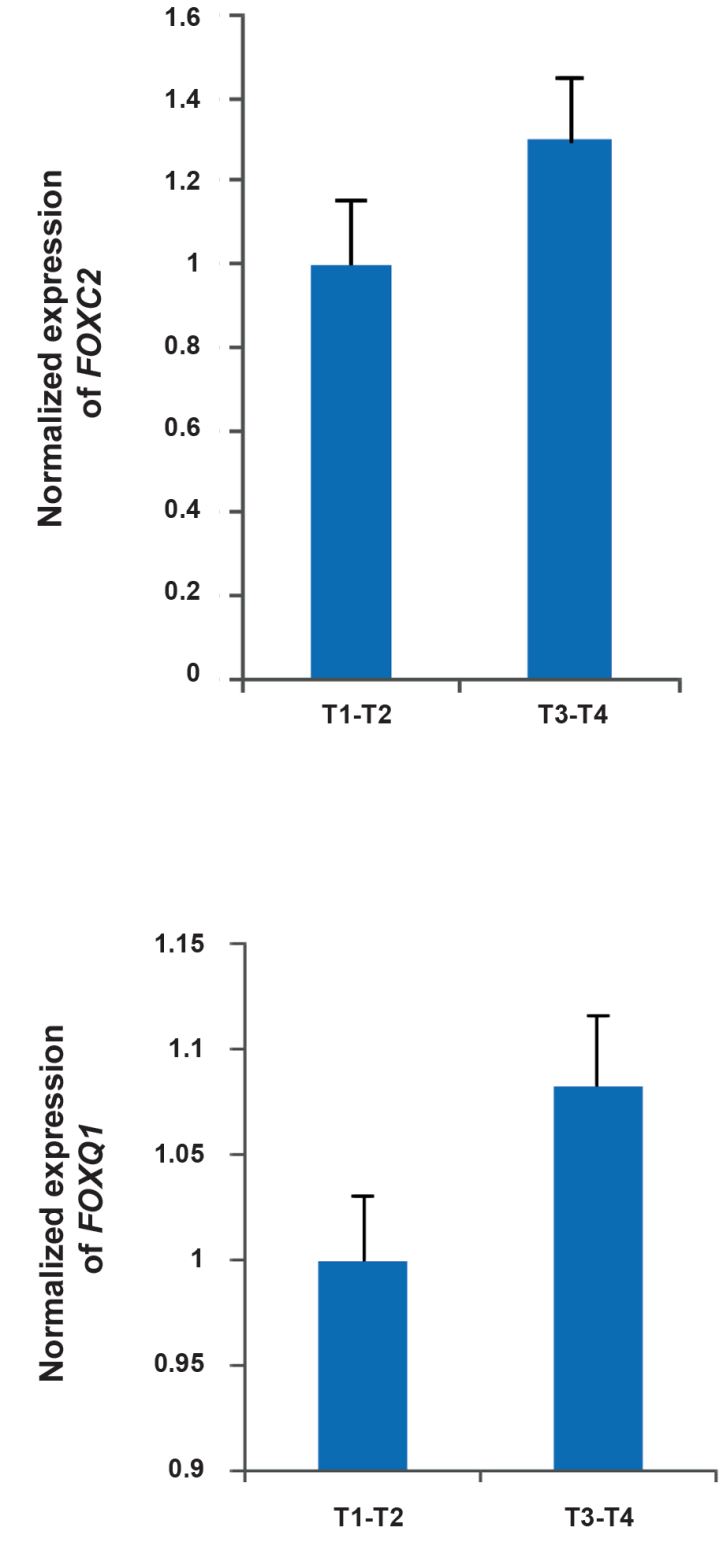
The expression of *FOXC2* and *FOXQ1* is not significantly different between different stages in gastric adenocarcinoma tissues. T1, 2, 3, 4; Different stage of tumor.

*FOXC2* and *FOXQ1* were not significantly differentially expressed between high (T3-T4)- and low (T1-T2)-stage tumors (Fig.3).

## Discussion

Different members of the forkhead box (*FOX*) family of transcription factors regulate various biological processes including cellular development and differentiation (19). Based on this, multiple studies have identified their crucial role in the progression of different cancers. In this study, we have shown the potential role of *FOXC2* and *FOXQ1* in the progression of gastric adenocarcinoma. The results indicate that upregulation of these genes is associated with higher grades gastric tumors.

A number of studies have shown that *FOXC2* expression is directly associated with EMT and cancer stem cell properties including disease recurrence, drug resistance, cell invasion, metastasis and poor prognosis (7, 8, 10). *FOXC2* specifically promotes mesenchymal differentiation during the EMT and is associated with cancer metastasis in aggressive basal-like breast cancers (3, 6). Interestingly, *FOXC2* expression is induced by a large number of known regulators of EMT, notably the Twist, Snail, and Goosecoid transcription factors as well as transforming growth factor-beta1 (TGF-β1). This suggests that *FOXC2* is involved in a diverse array of EMT programs (20). Zhu et al. (13), in a study on gastric tumors, found that *FOXC2* has higher expression at the protein level. The immunohistochemistry results showed that this protein can be a potential biomarker for gastric cancer. This is consistent with results herein showing that *FOXC2* has also significantly higher transcript levels in high grade gastric tumors. Previous studies have also described a key role for *FOXQ1* in regulating EMT and aggressiveness in human cancer (10, 12, 14). Unlike *FOXC2*, which does not seem to affect E-cadherin transcription, *FOXQ1*-induced EMT is accompanied by transcriptional repression of E-cadherin (21).

*FOXQ1* transcript is highly expressed in murine tissues, particularly in stomach and bladder (22). Recently, it has been reported that it plays a role in stomach surface cells. This is because *FOXQ1*- deficient mice exhibit a lack of gastric acid secretion in response to various stimuli (3, 22). Furthermore, mucin expression and granule content in mucous cells of mouse stomach surface is also shown to be under the control of *FOXQ1* (22). With respect to the important role of *FOXQ1* in gastric development, we investigated its role in gastric tumorigenesis. We show that its up-regulation is associated with high-grade gastric tumors, thus indicating that abundance of its encoded protein may aid gastric cancer progression. Liang et al. (15), in a similar study, also found the up-regulation of *FOXQ1* in gastric tumors at both transcript and protein levels. The current study was done on more samples and supports the potential role of *FOXQ1* in gastric carcinogenesis.

The origin of gastric adenocarcinoma, which is a malignant epithelial tumor, is from the granular epithelium of the gastric mucosa. Studies have shown that the aberrant activation of EMT has an impact on adult epithelial cancer development (23). Since *FOXC2* and *FOXQ1* seem to be involved in EMT progression, we hypothesize that overexpression of these genes may be attributed to their role in regulation of EMT (24).

## Conclusion

We demonstrate that *FOXC2* and *FOXQ1* are both associated with gastric cancer progression. Therefore, both may potentially be used as targets for prognosis of patients. Nevertheless, further investigation should be done for it to reach clinical trials.
